# Identification of Nutmeg With Different Mildew Degree Based on HPLC Fingerprint, GC-MS, and E-Nose

**DOI:** 10.3389/fnut.2022.914758

**Published:** 2022-06-28

**Authors:** Rui-Qi Yang, Jia-Hui Li, Hui-Shang Feng, Yue-Bao Yao, Xing-Yu Guo, Shu-Lin Yu, Yang Cui, Hui-Qin Zou, Yong-Hong Yan

**Affiliations:** ^1^School of Chinese Materia Medica, Beijing University of Chinese Medicine, Beijing, China; ^2^Department of Dermatology, Dongzhimen Hospital Beijing University of Chinese Medicine, Beijing, China

**Keywords:** nutmeg, mildew, electronic nose, GC-MS, HPLC fingerprint

## Abstract

Nutmeg (*Myristicae* Semen), the so-called *Rou-Dou-Kou* in Chinese, is one kind of Chinese herbal medicines (CHMs) as well as a globally popular spice. Hence, its stable quality and safe application attract more attention. However, it is highly prone to mildew during storage due to its rich volatile components and fatty oil. Therefore, in this study, an electronic nose (E-nose) was introduced to attempt to reliably and rapidly identify nutmeg samples with different degrees of mildew. Meanwhile, the chemical composition and volatile oil were analyzed using HPLC fingerprint and GC-MS, respectively, which could support and validate the result of E-nose. The results showed that the cluster results of HPLC fingerprint and GC-MS were generally consistent with E-nose, and they all clustered into two categories. Additionally, a discriminant model was established, which divided the samples into three categories: mildew-free, mildew-slight, and mildew, and a high DPR was obtained, which indicates that the E-nose could be a novel and promising approach for the establishment of a quality evaluation system to identify CHMs with different degrees of mildew rapidly, especially to identify early mildew.

## Introduction

Nutmeg (*Myristicae* Semen), the so-called *Rou-Dou-Kou* in Chinese, is not only one of the commonly applied herbs in CHMs for more than 1,200 years, mainly for the treatment of gastrointestinal diseases, but also used as a food additive in many countries, with an annual consumption of more than 500,000 tons. However, nutmeg is highly prone to mildew during storage due to its rich volatile components and fatty oil, which could reduce or lose its efficacy and even produce mycotoxins. For instance, the aflatoxins intake of even minor concentrations such as 1.0 ng/kg bw/day is considered toxic to humans (1), and its major health implication is liver cancer (2). At present, the main method for distinguishing the mildew of nutmeg is the macroscopic identification based on artificial sensory evaluation. During the experiment, it is almost impossible to ensure whether the nutmeg gets mildew or not without cutting it apart. Moreover, it is difficult to identify the degree of mildew, especially after samples have been ground to powder.

Therefore, it should not be overlooked that the obvious drawbacks of macroscopic identification are strong subjectivity, poor repeatability, and highly dependent on individual human perceptions. Nevertheless, the existing physical and chemical detection methods are generally complicated, expensive, time-consuming, and non-environment-friendly. In addition, the components of CHMs are complex; as a result, it is inevitable that there are limitations in determining the quality of CHMs only by determining one or several chemical compositions.

Then, the key question is as follows: Is there a simple, quick, and comprehensive way to identify nutmeg samples with different degrees of mildew? At the initial stage of moldy, nutmeg has been identified as qualified, but its smell has already changed slightly, which is difficult to perceive by the human senses.

In recent years, electronic nose (E-nose) technology based on sensitive and objective gas-sensing sensor array and pattern recognition technology has been applied to the detection of food flavor (3), meat freshness (4), pest in agriculture (5), quality evaluation of tobacco (6), and so on. Besides, our team has been working on the application of E-nose in the quality evaluation of CHMs for more than 17 years, with impressive and reliable achievements. For example, E-nose was introduced to establish the classification model of eight species of Asteraceae plants combined with a radial basis function (RBF) artificial neural network (7). E-nose combined with linear discriminant analysis and RBF neural network could correctly classify *L. japonica* samples of different storage months (8). A total of 20 batches of Amomi Fructus were analyzed based on organoleptic evaluation, E-nose, and GC-MS, and the results demonstrated that E-nose and GC-MS analysis were in great agreement with organoleptic evaluation (9), demonstrating the ability of E-nose in the digitalization of CHMs odor and bringing us a promising tool for rapid identification and online detection of CHMs quality.

In this study, first the odor information of nutmeg samples was obtained using E-nose, while the chemical components were analyzed using HPLC fingerprint and GC-MS, respectively. And then hierarchical cluster analysis (HCA) was utilized to analyze the three results and compare the similarities and differences of the clustering results. Finally, the E-nose discrimination model was established by three classifiers [naive-Bayes network (NBN), radial basis function network (RBF), and random forest (RF)] to prove the applicability of the E-nose in CHMs mildew discrimination. The Bayesian classification algorithm is a statistical classification method, which is a class of algorithms using probabilistic statistical knowledge for classification. NBN is a simple and efficient classifier, widely used in data mining and pattern recognition (10). RBF networks are well-known for their ability to solve complex non-linear problems with single-layered neural networks in recent years, and it has witnessed a number of successful applications, such as classification, regression, system identification, and pattern recognition (11). RF is a classifier consisting of many decision trees, and each of these trees is a classifier; it has proven to be an effective classification method in many fields, as it is more accurately and reliably than a single classifier (12).

All in all, this study aims to (1) establish a discriminative model based on odor fingerprints and an artificial network for rapid differentiation of the mildew degrees of different nutmeg samples, (2) provide a method and solid experimental data for pre-detection and even an early-warning for CHMs with the potential exogenous toxin.

## Materials and Methods

### Chemicals and Reagents

Methanol and other reagents used were all of chromatographic grade and were purchased from Thermo Fisher Technology (China) Co., Ltd. (Shanghai, China).

### Nutmeg Samples

Nutmeg samples were purchased from Hebei Xinghua Traditional Chinese Medicine Co., Ltd. (Heibei, China) in January 2018 and authenticated based on macroscopical identification (which is one of the methods of the *Chinese Pharmacopeia*) and identified by observing the surface and cut texture of nutmeg and smelling the taste of nutmeg through the nose by Professor Yong-Hong Yan from Department of Chinese Materia Medica of Beijing University of Chinese Medicine. Samples with different degrees of mildew were prepared by a constant temperature and humidity accelerated test (30°C, 95% RH, protected from light). Samples were taken every 3 days, and 27 batches of samples were obtained by accelerated 78 days, which were numbered from J0 to J26.

### HPLC Fingerprint Analysis

Nutmeg samples were first ground and sifted through a mesh of 850 μm for a fine and unified powder. Then, 0.2 g of powder was accurately weighed into 50 ml conical flasks, followed by precisely added 25 ml of methanol and extracted with ultrasonic at 40 kHz for 30 min. After cooling down to the room temperature and compensating weight, the processed samples were filtered and stored at 4°C before analysis.

The HPLC system includes a Waters e2695 instrument with an ultraviolet detector (UVD). HPLC conditions: ZORBAX SB-C18 column (4.6 mm × 250 mm × 5 μm). Mobile phase A: water, C: methanol; gradient elution (13) was used from 40% A to 35% A for 10 min, from 35% A to 30% A for 15 min, from 30% A to 22% A for 10 min, from 22% A to 20% A for 10 min, from 20% A to 15% A for 5 min, from 15% A to 10% A for 10 min; detection wavelength: 274 nm; column temperature: 25°C; and injection volume: 10 μl.

### GC-MS Analysis of Volatile Oil

Nutmeg samples were first ground and sifted through a mesh of 850 μm, 20 g of powder was accurately weighed into 1,000 ml round-bottom flask, and precisely added 500 ml of pure water. Then extracted the volatile oil according to the general rule 2204 volatile oil determination method (method a) of the 2015 edition of the *Chinese Pharmacopeia* (14). After being dried with anhydrous sodium sulfate, 0.1 g of volatile oil was accurately weighed into a 5 ml volumetric flask and diluted with petroleum ether to the scale. The processed samples were filtered and stored at 4°C until analysis.

The GC-MS system includes an Agilent 7890B instrument with a 5977A mass spectrum detector (MSD). GC-MS conditions: Agilent19091S-433HP-5MS column (30 m × 250 μm × 0.25 μm); inlet temperature: 230°C; column oven temperature: initial temperature 60°C, keep for 1 min; rise to 70°C at the rate of 5°C/min, keep for 5 min; rise to 160°C at the rate of 5°C/min, keep for 5 min; rise to 200°C at the rate of 6°C/min, keep for 1 min; carrier gas: helium; flow rate: 1 ml/min; split ratio: 25:1; injection volume: 1 μl; interface temperature: 260°C; ionization mode: EI; electronic energy: 70 eV; ion source temperature: 230°C; quadrupole temperature: 150°C; solvent delay: 3 min; tuning mode: standard tuning; and mass scanning range: 30–500 amu.

### E-Nose Analysis

α-Fox3000 E-nose (Alpha MOS, Co., Ltd., France) with 12 metal-oxide gas sensors was employed to obtain the digitalized odor fingerprint. This E-nose instrument consists of an automatic sampling system, namely, HS-100 sampler, two chambers with six sensors in each, and a computer for data acquisition. Each of the total 12 sensors has different detection sensitivity, as shown in Table 1. The response value of E-nose is expressed as the ratio of conductance: G_0_/G (where G_0_ represents the conductance of a sensor in the reference air, while G represents the conductance of the sensor in the sample gas).

**Table 1:** Main application of 12 sensors in α-Fox3000 E-nose.

**Number**	**Sensor name**	**Main application**
S1	LY2/LG	Oxidizing gas
S2	LY2/G	Ammonia, carbon monoxide
S3	LY2/AA	Ethanol
S4	LY2/GH	Ammonia/organic amine
S5	LY2/gCTL	Hydrogen sulfide
S6	LY2/gCT	Propane/butane
S7	T30/1	Organic solvents
S8	P10/1	Hydrocarbons
S9	P10/2	Methane
S10	P40/1	Fluorine
S11	T70/2	Aromatic compounds
S12	PA/2	Ethanol, ammonia/organic amine

Nutmeg samples were first ground and sifted through a mesh of 850 μm and then accurately weighed 0.2 g in 10 ml headspace vials. According to the optimal results of the pre-experiment, the incubation conditions were programmed at 35°C for 60 s with a rotating speed of 250 r/min. The temperature and volume of injection were set to 45°C and 300 μl, respectively. As the carrier gas, the flow rate of pure air was set at 150 ml/min. The sampling time was set to 120 s, the sampling interval was set to 1 s, and the cleaning time of the sensor array was set to 1,080 s. Every sample was continuously sampled seven times.

### Statistical Analysis

To compare the similarities and differences of E-nose, HPLC fingerprint, and GC-MS results, HCA analysis using SAS 8.2 was carried out, and then the HCA analysis results were compared with a color table, which could present the clear and direct results.

For HPLC fingerprint, similarity evaluation system of CHMs chromatographic fingerprint (version 2012.130723) was used to match the chromatographic peaks. And then HCA was utilized to cluster these peaks (if there were no peaks in the retention time, the peak area was recorded as 0). For GC-MS, compounds were identified using the NIST14.L library search data system (the matching degree was more than 85%) and reference-related literature. The quantification of each compound was carried out by peak area normalization. As mentioned above, HCA was utilized to cluster the relative content of volatile oil. For E-nose, the maximum response value of each sensor was chosen as a feature value for data analysis.

To prove the applicability of the E-nose in CHMs mildew discrimination, discriminant analysis of the E-nose data was performed using the Origin (version 8.5) software. Then, three kinds of classifiers, namely, NBN, RBF, and RF, were used to establish the discriminant models, respectively. Finally, 10-fold cross-validation and external test set validation were used to evaluate the performance of different classifiers with the differential positive rate (DPR). The classification results should not be accepted if the DPR was lower than 80% (15).

## Results and Discussion

### HPLC Fingerprint Analysis Results

**Figure 1:**
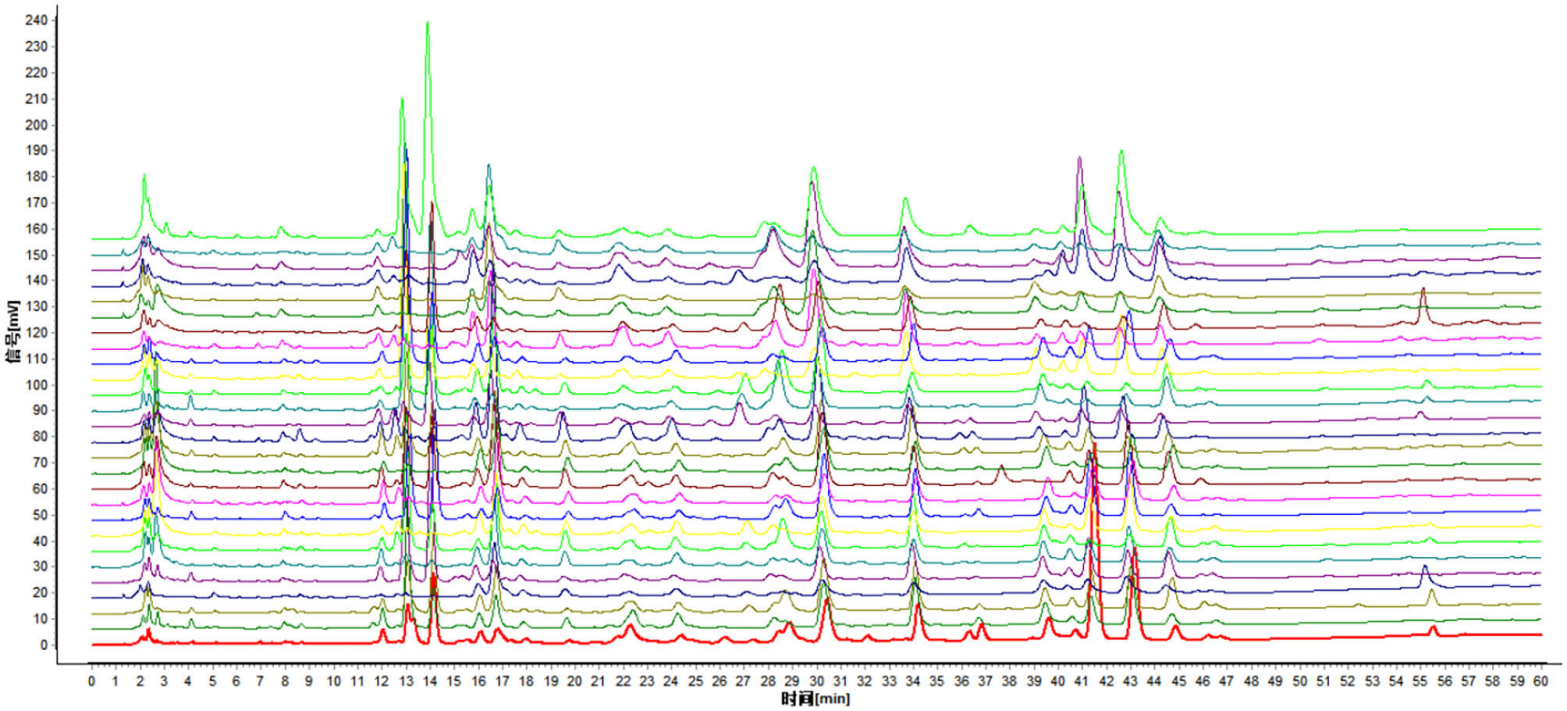
HPLC fingerprint overlay of 27 batches of nutmeg samples.

A total of 27 batches of samples were matched by the similarity evaluation system of CHMs chromatographic fingerprint. Taking the J0 sample chromatogram as a reference, a total of 93 peaks were obtained, of which only 7 were common peaks, and compared with the HPLC fingerprints of J0 samples, the similarity of the other 26 batches of samples were <80%. This indicated that the chemical composition of the nutmeg had changed dramatically before and after getting moldy. The fingerprint overlay of the samples is shown in Figure 1, from bottom to top are samples J0 to J26 in order.

**Figure 2:**
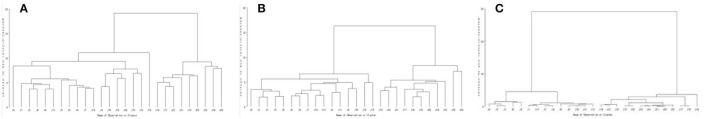
Clustering result of HPLC fingerprint data **(A)**, volatile oils of GC-MS **(B)**, and E-nose **(C)**.

According to the existence and size of peak area (if there is no chromatographic peak at this retention time, the peak area is recorded as 0), HCA based on ward method was utilized to analyze these matched 93 peaks. It was found that all samples were classified into two major categories and six subcategories as demonstrated in Figure 2A, before J13 (including J13), the clustering categories were mainly mildew-slight samples, and the subsequent clustering categories (i.e., after J14) were mainly mildew samples. Combined with similarity analysis, the similarity of J13 and J14 samples was only 0.15, and the proportion of chemical components in the samples is significantly different, so it may become the critical point of classification.

### GC-MS Analysis Result

Since the volatile oil is one of the main chemical components in nutmeg, GC-MS has utilized to analyze the compounds of it, which aimed to reveal the varying material basis of volatile oil in nutmeg samples during the mildew process. The composition details of the volatile oils are shown in Supplementary Table 1 (due to limited space, only the information on even-numbered sample is listed). A total of 44 compounds were identified in all samples, including 35 common compounds, which are mainly composed of terpenes, phenylpropenes, and alcohols. It is worth noting that the volatile oil content of nutmeg samples after mildew was still not lower than the standard of *Chinese Pharmacopeia*, but the composition of volatile oil changed dramatically. Compared with the percentage change of each component, it was found that with the extension of acceleration time, the content of myristic ether and element, which have a hallucinogenic effect, tended to increase.

According to the relative content (the relative percentage content of the peak area normalized) of each component, HCA based on the ward method was utilized to analyze these 44 components. It was found that all samples were classified into two major categories as shown in Figure 2B. It is illustrated in Figure 2B that the clustering of J0–J26 samples is relatively compact, indicating that the effect of mildew on volatile oil content is a slow process.

### HCA Analysis Result of E-Nose

Based on seven parallel measurements of samples, the relative standard deviation (RSD, *n* = 7) of the maximum response value of each sensor was calculated, the results were all <3%, proving that the experiment has good repeatability.

HCA based on ward method was utilized to analyze the maximum response value of E-nose. As shown in Figure 2C, all samples were classified into two major categories. Comparing the cluster results of HPLC fingerprint, GC-MS, and E-nose, it was found that the classification of mildew degree of J0–J13 samples (identified as mildew- slight) was consistently classified by the three methods. However, the classification of J14–J26 samples (identified as mildew) were slightly different, samples J15, J16, J18, and J20 of HPLC fingerprint and J15 and J16 of GC-MS were classified as mildew-slight, which may be due to the individual differences of nutmeg samples. The changes of chemical composition during mildewing were slow, and the speed of changes was different in each nutmeg sample, while the E-nose could more sensitively capture the changes in odor. In general, the clustering result of E-nose is generally consistent with those of HPLC fingerprint and volatile oil GC-MS, and J14 samples as the demarcation point, suggesting that the composition of samples changes at this stage (i.e., accelerating 42–48 days) and thus entered the mildew state. The consistency of the clustering results indicates that the odor changes detected by the E-nose are somewhat associated with intrinsic volatile and non-volatile substances. However, the J0 sample is not a mildew sample, clustered with a slightly mildew sample, indicating that mildew deterioration is a slow process, and its chemical composition and smell changes are very small.

### Discriminant Models of E-Nose

**Figure 3:**
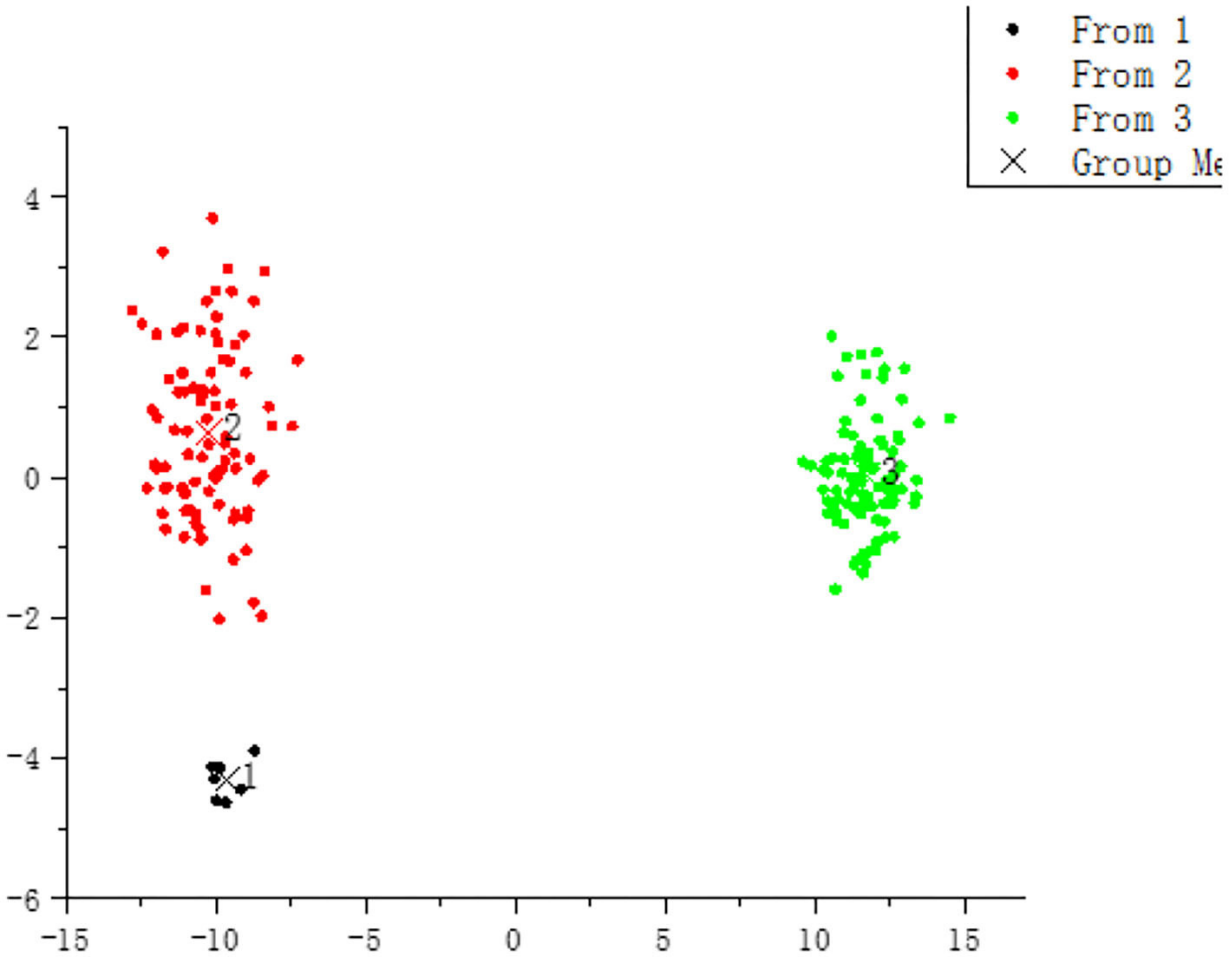
Discriminant models of E-nose.

As shown in Figure 3, all samples were divided into three groups by the discriminant analysis. Table 2 exhibits the DPR values of the three classifiers evaluated by the 10-fold cross-validation and external test set validation. The DPR values of three classifiers were far more than 85%, indicating that the E-nose could distinguish nutmeg samples with different degrees of mildew. Combined with macroscopic identification, samples No. 1 (black dots) was identified as mildew-free samples, No. 2 (red dots) as mildew-slight samples, and No. 3 (green dots) as mildew samples.

**Table 2:** DPR of three classifiers (NBN, RBF, and RF).

**Type of classifier**	**Tenfold cross validation**	**External test set validation**
Native bayes net	98.46	94.91
RBF network	99.48	100
Random forest	100	96.61

The E-nose discrimination model is consistent with the result of macroscopic identification; however, the latter one often requires identification subject with extensive experience and integral observation object. Even so, it is impossible to ensure the reliability and repetition of the identification result, as it has a high subjective will. Then, the E-nose not only could make up for the above shortcomings, it is objective and convenient, without high requirements for the identification of the subject and object, but it could also sensitively capture or even early warn the changes of subtle odor, which represent the intrinsic trace volatile components.

In recent years, E-nose has been applied to the precise prediction of the chemical composition content in many fields. E-nose is combined with ANN modeling for qualitative and quantitative analysis of benzoic acid in cola-type beverages (16). E-nose cooperates with other analysis methods for quantitative analysis of baicalin and other components in *Xiao-chai-hu* granules (17). In this study, the intrinsic composition is calculated using the relative content, and in subsequent studies, we will determine the absolute content of the components with clear pharmacological effects and correlate it with the E-nose results to explore the correlation of response values and chemical composition.

## Conclusion

For quick and reliable identification of the mildew state of nutmeg, 27 batches of nutmeg samples with different degrees were prepared by accelerated test, and E-nose was introduced to obtain the odor information for rapid and reliable identification. To verify the consistency of the intrinsic composition changes with the E-nose detection results, HPLC fingerprint and GC-MS were carried out to analyze the chemical composition and volatile oils, respectively. The results showed that the cluster results of HPLC fingerprint and GC-MS analysis were generally consistent with E-nose, although there were some acceptable errors, which reveals that the odor changes detected by the E-nose are somewhat associated with intrinsic volatile and non-volatile substances, and in turn, the changes of internal components also support and supplement the identification model of E-nose. Additionally, discriminant model was established to prove the applicability of the E-nose in CHMs mildew discrimination, and a high DPR was obtained, which indicate that the E-nose can be used as a new quality evaluation direction to identify CHMs with different degrees of mildew.

Our team is committing to the accurate prediction of effective and harmful components in CHMs by E-nose and realized the rapid and comprehensive evaluation of the safety and effectiveness of CHMs. One of the studies was to combine E-nose and HS-GC-MS to explore the correlation between E-nose sensors and volatile components in the process of nutmeg mildew, and the results showed that the compounds of aldehyde, alcohol, and ester produced in the mildew process could be the physical basis for flavor change and moldy odor production (18). Based on these numerous E-nose researches in CHMs, we wish that the future development direction of sensors can be oriented toward exclusive research and development of rapid identification of materials.

However, at present, no electronic sensor is specially designed for components of CHMs. We hope that researchers in machinery or other related fields could design an E-nose sensor array focusing on detecting the chemical components of CHMs to achieve more accurate prediction.

## Data Availability Statement

The raw data supporting the conclusions of this article will be made available by the authors, without undue reservation.

## Author Contributions

R-QY analyzed the data and prepared the manuscript and arranged all experiments in this study. R-QY and J-HL carried out the volatile oil extraction and GC-MS experiments. R-QY and Y-BY performed the samples collection and preparation. R-QY and X-YG conducted the HPLC fingerprint experiments. R-QY, S-LY, and YC conducted the E-nose experiments and data analysis. H-SF provided clinical medication guidance. H-QZ and Y-HY designed the study, provided theoretical guidance, and administrated the projects. H-QZ revised the manuscript. All authors contributed to the article and approved the submitted version.

## Funding

This study was financially supported by the National Natural Science Foundation of China (Grant Nos. 81573542 and 81403054), the Youth Teachers Project of Beijing University of Chinese Medicine (Grant No. 2019-JYB-JS-006), and the Independent Selection Project for Postgraduates of Beijing University of Chinese Medicine (Grant No. 2018-JYB-XS073).

## Conflict of Interest

The authors declare that the research was conducted in the absence of any commercial or financial relationships that could be construed as a potential conflict of interest.

## Publisher's Note

All claims expressed in this article are solely those of the authors and do not necessarily represent those of their affiliated organizations, or those of the publisher, the editors and the reviewers. Any product that may be evaluated in this article, or claim that may be made by its manufacturer, is not guaranteed or endorsed by the publisher.
