# Supplementary material for: Identification of Nutmeg With Different Mildew Degree Based on HPLC Fingerprint, GC-MS, and E-Nose
Source: Front Nutr. 2022 Jun 28;9:914758. doi: 10.3389/fnut.2022.914758 (PMC9274197; doi:10.3389/fnut.2022.914758)
Supplement: Supplementary file 1 [file Table_1.DOCX]

**Supplementary Information**

Table 1-1. Identified volatile compounds of nutmeg by GC-MS (relative content %)

| No. | Category | Compound | J0 | J1 | J2 | J3 | J4 | J5 | J6 | J7 | J8 | J9 | J10 | J11 | J12 | J13 | J14 |
| --- | --- | --- | --- | --- | --- | --- | --- | --- | --- | --- | --- | --- | --- | --- | --- | --- | --- |
| 1 | Terpenes | alpha.-Pinene | 9.99 | 9.46 | 10.54 | 11.07 | 12.61 | 9.85 | 10.03 | 12.09 | 9.54 | 10.62 | 10.21 | 10.19 | 11.65 | 10.95 | 8.68 |
| 2 |  | Camphene | 0.19 | 0.17 | 0.19 | 0.20 | 0.25 | 0.20 | 0.18 | 0.22 | 0.17 | 0.21 | 0.18 | 0.18 | 0.21 | 0.21 | 0.15 |
| 3 |  | Bicyclo[3.1.0]hexane, 4-methylene-1-(1-methylethyl)- | 25.78 | 23.15 | 25.19 | 24.43 | 23.59 | 23.63 | 24.37 | 21.9 | 20.72 | 23.29 | 21.73 | 20.73 | 20.32 | 22.36 | 16.43 |
| 4 |  | Bicyclo[3.1.1]heptane, 6,6-dimethyl-2-methylene-, (1S)- | 8.09 | 7.3 | 8.08 | 8.49 | 9.56 | 7.35 | 7.53 | 9.93 | 6.80 | 8.48 | 7.63 | 7.36 | 8.94 | 8.71 | 7.38 |
| 5 |  | beta-Myrcene | 2.32 | 2.01 | 2.18 | 2.22 | 2.30 | 2.16 | 2.18 | 2.2 | 1.94 | 2.15 | 2.10 | 2.03 | 2.11 | 2.39 | 1.73 |
| 6 |  | alpha.-Phellandrene | 0.81 | 0.58 | 0.61 | 0.64 | 0.55 | 0.73 | 0.61 | 0.55 | 0.52 | 0.61 | 0.65 | 0.71 | 0.62 | 0.92 | 0.56 |
| 7 |  | 3-Carene | 0.93 | 0.33 | 0.51 | 0.46 | 0.39 | 0.54 | 0.51 | 0.38 | 0.29 | 0.53 | 0.74 | 0.48 | 0.52 | 0.43 | 0.33 |
| 8 |  | (+)-4-Carene | 2.00 | 2.08 | 1.85 | 2.22 | 2.28 | 2.10 | 2.08 | 2.29 | 2.45 | 2.28 | 2.42 | 2.56 | 2.56 | 2.86 | 2.33 |
| 9 |  | D-Limonene | 7.20 | 5.96 | 6.12 | 6.25 | 6.02 | 7.58 | 6.22 | 6.02 | 5.58 | 6.09 | 5.75 | 6.54 | 6.04 | 9.36 | 5.99 |
| 10 |  | Eucalyptol | 0.17 | 0.16 | 0.16 | 0.16 | 0.18 | 0.18 | 0.16 | 0.16 | 0.15 | 0.16 | 0.15 | 0.17 | 0.16 | 0.20 | 0.12 |
| 11 |  | gamma.-Terpinene | 3.25 | 3.36 | 3.02 | 3.57 | 3.70 | 3.37 | 3.43 | 3.71 | 4.00 | 3.79 | 3.99 | 4.26 | 4.28 | 4.74 | 4.18 |
| 12 |  | Cyclohexene, 1-methyl-4-(1-methylethylidene)- | 1.33 | 1.02 | 1.07 | 1.14 | 1.15 | 1.11 | 1.14 | 1.13 | 1.10 | 1.22 | 1.42 | 1.30 | 1.32 | 1.34 | 1.11 |
| 13 |  | Ylangene | 0.27 | 0.31 | 0.31 | 0.31 | 0.37 | 0.30 | 0.32 | 0.32 | 0.37 | 0.32 | 0.37 | 0.28 | 0.31 | 0.29 | 0.28 |
| 14 |  | alfa-Copaene | 0.52 | 0.61 | 0.55 | 0.62 | 0.64 | 0.66 | 0.72 | 0.53 | 0.78 | 0.53 | 0.80 | 0.52 | 0.55 | 0.72 | 0.73 |
| 15 |  | Caryophyllene | 0.59 | 0.5 | 0.49 | 0.23 | 0.29 | 0.20 | 0.28 | 0.51 | 0.31 | 0.18 | 0.20 | 0.19 | 0.27 | 0.61 | 0.24 |
| 16 |  | cis-.alpha.-Bergamotene | 0.15 | 0.15 | 0.16 | 0.18 | 0.20 | 0.14 | 0.16 | 0.13 | 0.16 | 0.13 | 0.14 | 0.15 | 0.13 | 0.14 | 0.10 |
| 17 |  | (E)-.beta.-Farnesene | 0 | 0 | 0 | 0 | 0.16 | 0.15 | 0.17 | 0.22 | 0.15 | 0.15 | 0.12 | 0.11 | 0.14 | 0 | 0.14 |
| 18 |  | Germacrene D | 0.23 | 0.33 | 0.23 | 0.20 | 0.17 | 0.27 | 0.33 | 0.17 | 0.23 | 0.2 | 0.27 | 0.20 | 0.21 | 0.30 | 0.39 |
| 19 |  | beta.-Bisabolene | 0.17 | 0.19 | 0.16 | 0.15 | 0.20 | 0.16 | 0.18 | 0.15 | 0.19 | 0.16 | 0.18 | 0.19 | 0.14 | 0.16 | 0.11 |
| 20 | Olefin | Cyclohexene-2-ethenly-1,3,3-trimethyl- | 0 | 0 | 0 | 0 | 0 | 0 | 0 | 0 | 0 | 0 | 0 | 0 | 0 | 0 | 0 |
| 21 | Phenylpropenes | Safrole | 1.75 | 2.41 | 2.39 | 2.36 | 2.46 | 1.92 | 2.57 | 2.91 | 2.27 | 1.61 | 2.80 | 1.30 | 2.22 | 2.74 | 2.81 |
| 22 |  | Eugenol | 0.18 | 0.29 | 0.24 | 0.23 | 0.24 | 0.22 | 0.24 | 0.26 | 0.21 | 0.16 | 0.24 | 0.20 | 0.27 | 0.19 | 0.14 |
| 23 |  | Methyleugenol | 5.64 | 7.88 | 7.88 | 5.71 | 5.81 | 6.59 | 6.16 | 4.64 | 11.72 | 7.73 | 5.00 | 12.89 | 6.01 | 3.49 | 10.78 |
| 24 |  | trans-Isoeugenol | 0.29 | 0.32 | 0.19 | 0.26 | 0.46 | 0.33 | 0.43 | 0.31 | 0.26 | 0.25 | 0.26 | 0.23 | 0.35 | 0.24 | 0.15 |
| 25 |  | Benzene, 1,2-dimethoxy-4-propenyl-, (Z)- | 3.09 | 2.82 | 2.73 | 0.68 | 1.14 | 2.00 | 2.02 | 0.76 | 2.82 | 2.45 | 2.30 | 5.69 | 1.16 | 0.84 | 2.20 |
| 26 |  | 1,3-Benzodioxole, 4-methoxy-6-(2-propenyl)- | 10.09 | 12.75 | 11.56 | 12.07 | 9.58 | 12.73 | 13.56 | 13.24 | 11.45 | 10 | 14.75 | 6.35 | 13.34 | 8.52 | 13.18 |
| 27 |  | Benzene,1,2,3-trimethoxy-5-(2-propenyl)- | 3.79 | 4.81 | 3.03 | 4.02 | 3.48 | 3.45 | 2.91 | 3.2 | 2.70 | 3.71 | 3.12 | 1.76 | 3.16 | 1.70 | 6.27 |
| 28 |  | Phenol, 2,6-dimethoxy-4-(2-propenyl)- | 0 | 0 | 0 | 0 | 0 | 0 | 0 | 0 | 0 | 0 | 0 | 0 | 0 | 0 | 0 |
| 29 | Alkanes | Bicyclo[3.1.0]hex-2-ene, 4-methyl-1-(1-methylethyl)- | 1.74 | 1.72 | 1.61 | 1.92 | 2.04 | 1.85 | 1.66 | 2.04 | 2.01 | 2.08 | 1.92 | 2.09 | 2.05 | 2.31 | 1.66 |
| 30 | Alcohols | Bicyclo[3.1.0]hexan-2-ol, 2-methyl-5-(1-methylethyl)-, (1.alpha.,2.beta.,5.alpha.)- | 0.35 | 0.34 | 0.44 | 0.33 | 0.28 | 0.29 | 0.34 | 0.22 | 0.30 | 0.49 | 0.27 | 0.43 | 0.33 | 0.68 | 0.39 |
| 31 |  | Bicyclo[3.1.0]hexan-2-ol, 2-methyl-5-(1-methylethyl)-, (1.alpha.,2.alpha.,5.alpha.)- | 0.29 | 0.28 | 0.37 | 0.25 | 0.20 | 0.22 | 0.28 | 0.16 | 0.24 | 0.40 | 0.21 | 0.36 | 0.26 | 0.6 | 0.32 |
| 32 |  | Linalool | 0.28 | 0.23 | 0.27 | 0.24 | 0.30 | 0.29 | 0.27 | 0.23 | 0.23 | 0.19 | 0.23 | 0.24 | 0.24 | 0.22 | 0.20 |
| 33 |  | 4-Isopropyl-1-methylcyclohex-2-enol | 0.41 | 0.41 | 0.39 | 0.45 | 0.42 | 0.43 | 0.41 | 0.4 | 0.46 | 0.45 | 0.43 | 0.47 | 0.44 | 0.56 | 0.42 |
| 34 |  | 2-Cyclohexen-1-ol, 1-methyl-4-(1-methylethyl)-, cis- | 0.27 | 0.27 | 0.25 | 0.29 | 0.42 | 0.29 | 0.27 | 0.26 | 0.30 | 0.28 | 0.28 | 0.30 | 0.28 | 0.35 | 0.26 |
| 35 |  | 3-Cyclohexen-1-ol, 4-methyl-1-(1-methylethyl)-, (R)- | 5.94 | 6.17 | 5.57 | 6.76 | 6.72 | 6.51 | 6.44 | 6.67 | 7.38 | 6.92 | 7.16 | 7.52 | 7.43 | 8.30 | 8.02 |
| 36 |  | alpha.-Terpineol | 0.60 | 0.57 | 0.56 | 0.62 | 0.63 | 0.61 | 0.58 | 0.65 | 0.61 | 0.60 | 0.61 | 0.63 | 0.67 | 0.71 | 0.62 |
| 37 |  | 2-Cyclohexen-1-ol, 3-methyl-6-(1-methylethyl)-, cis- | 0.14 | 0.15 | 0.13 | 0.15 | 0.15 | 0.12 | 0.15 | 0.14 | 0.12 | 0.15 | 0.11 | 0.11 | 0.11 | 0.13 | 0.09 |
| 38 |  | 2-Cyclohexen-1-ol, 3-methyl-6-(1-methylethyl)-, trans- | 0 | 0 | 0 | 0 | 0 | 0.15 | 0 | 0 | 0.16 | 0 | 0.16 | 0.16 | 0.15 | 0.18 | 0.12 |
| 39 |  | 5-Azulenemethanol, 1,2,3,4,5,6,7,8-octahydro-.alpha.,.alpha.,3,8-tetramethyl- | 0 | 0 | 0 | 0 | 0 | 0 | 0 | 0 | 0 | 0 | 0 | 0 | 0 | 0 | 0 |
| 40 | Aromatic | p-Cymene | 0.91 | 0.72 | 0.73 | 0.85 | 0.83 | 1.13 | 0.93 | 0.88 | 0.97 | 0.88 | 0.89 | 0.89 | 0.86 | 1.33 | 0.90 |
| 41 |  | Benzene, 1-methoxy-4-pentyl- | 0 | 0 | 0 | 0 | 0 | 0 | 0 | 0 | 0 | 0 | 0 | 0 | 0 | 0 | 0 |
| 42 | Esters | Geranyl acetate | 0.27 | 0.20 | 0.26 | 0.26 | 0.23 | 0.17 | 0.19 | 0.25 | 0.16 | 0.24 | 0.23 | 0.20 | 0.22 | 0.20 | 0.16 |
| 43 |  | Tetradecanoi acid, ethyl ester | 0 | 0 | 0 | 0 | 0 | 0 | 0 | 0.14 | 0.21 | 0.31 | 0 | 0 | 0 | 0 | 0.31 |
| 44 | Fatty Acids | Tetradecanoic acid | 0 | 0 | 0 | 0 | 0 | 0 | 0 | 0 | 0 | 0 | 0 | 0 | 0 | 0 | 0 |

Table 1-2. Identified volatile compounds of nutmeg by GC-MS (relative content %)

| No. | Category | Compound | J15 | J16 | J17 | J18 | J19 | J20 | J21 | J22 | J23 | J24 | J25 | J26 |
| --- | --- | --- | --- | --- | --- | --- | --- | --- | --- | --- | --- | --- | --- | --- |
| 1 | Terpenes | alpha.-Pinene | 10.47 | 10.51 | 7.55 | 8.24 | 8.86 | 8.08 | 7.36 | 8.72 | 10.19 | 9.37 | 6.32 | 8.36 |
| 2 |  | Camphene | 0.18 | 0.19 | 0.14 | 0.16 | 0.17 | 0.16 | 0.13 | 0.17 | 0.20 | 0.17 | 0.12 | 0.14 |
| 3 |  | Bicyclo[3.1.0]hexane, 4-methylene-1-(1-methylethyl)- | 19.81 | 19.71 | 19.79 | 18.81 | 17.94 | 17.70 | 18.24 | 20.21 | 13.54 | 16.69 | 11.61 | 20.32 |
| 4 |  | Bicyclo[3.1.1]heptane, 6,6-dimethyl-2-methylene-, (1S)- | 10.28 | 8.89 | 6.70 | 6.92 | 7.11 | 6.90 | 6.49 | 7.07 | 8.11 | 7.61 | 6.29 | 5.99 |
| 5 |  | beta-Myrcene | 2.09 | 2.15 | 1.92 | 1.94 | 1.95 | 1.81 | 1.69 | 2.02 | 1.78 | 1.77 | 1.08 | 1.85 |
| 6 |  | alpha.-Phellandrene | 0.68 | 0.66 | 0.46 | 0.61 | 0.75 | 0.54 | 0.60 | 0.70 | 0.50 | 0.44 | 0.49 | 0.46 |
| 7 |  | 3-Carene | 0.59 | 0.22 | 0.36 | 0.43 | 0.48 | 0.41 | 0.55 | 0.34 | 0.33 | 0.29 | 0.24 | 0.20 |
| 8 |  | (+)-4-Carene | 2.52 | 2.74 | 2.87 | 2.64 | 2.44 | 2.67 | 2.19 | 2.36 | 2.26 | 2.65 | 2.34 | 2.64 |
| 9 |  | D-Limonene | 6.54 | 7.45 | 5.33 | 6.86 | 8.21 | 5.53 | 5.74 | 7.57 | 5.86 | 5.29 | 4.22 | 5.23 |
| 10 |  | Eucalyptol | 0.14 | 0.15 | 0.13 | 0.15 | 0.16 | 0.12 | 0.14 | 0.16 | 0.12 | 0.13 | 0.14 | 0 |
| 11 |  | gamma.-Terpinene | 4.54 | 4.83 | 5.12 | 4.73 | 4.38 | 4.81 | 3.98 | 4.10 | 3.94 | 4.97 | 4.41 | 4.60 |
| 12 |  | Cyclohexene, 1-methyl-4-(1-methylethylidene)- | 1.38 | 1.20 | 1.31 | 1.27 | 1.23 | 1.32 | 1.22 | 1.10 | 1.07 | 1.29 | 1.16 | 1.11 |
| 13 |  | Ylangene | 0.33 | 0.33 | 0.28 | 0.29 | 0.29 | 0.32 | 0.31 | 0.36 | 0.28 | 0.34 | 0.49 | 0.32 |
| 14 |  | alfa-Copaene | 0.6 | 0.83 | 0.69 | 0.60 | 0.51 | 0.82 | 0.63 | 0.94 | 0.62 | 0.66 | 0.99 | 0.74 |
| 15 |  | Caryophyllene | 0.69 | 0.19 | 0.62 | 0.42 | 0.23 | 0.27 | 0.33 | 0.68 | 0.36 | 0.11 | 0.45 | 0.39 |
| 16 |  | cis-.alpha.-Bergamotene | 0.16 | 0.23 | 0.19 | 0.17 | 0.15 | 0.17 | 0.14 | 0.18 | 0.15 | 0.13 | 0.09 | 0.18 |
| 17 |  | (E)-.beta.-Farnesene | 0 | 0.20 | 0 | 0 | 0 | 0.09 | 0 | 0.12 | 0.09 | 0.17 | 0.26 | 0.11 |
| 18 |  | Germacrene D | 0.47 | 0.25 | 0.24 | 0.21 | 0.19 | 0.26 | 0.30 | 0.28 | 0.22 | 0.23 | 0.39 | 0.24 |
| 19 |  | beta.-Bisabolene | 0.11 | 0.24 | 0.21 | 0.19 | 0.17 | 0.18 | 0.15 | 0.18 | 0.16 | 0.16 | 0.23 | 0.20 |
| 20 | Olefin | Cyclohexene-2-ethenly-1,3,3-trimethyl- | 0 | 0 | 0 | 0.05 | 0.10 | 0.11 | 0 | 0 | 0.08 | 0.11 | 0.14 | 0.37 |
| 21 | Phenylpropenes | Safrole | 1.59 | 1.67 | 2.28 | 1.77 | 1.33 | 1.78 | 3.08 | 1.34 | 1.95 | 2.32 | 1.84 | 1.41 |
| 22 |  | Eugenol | 0.14 | 0.25 | 0.19 | 0.18 | 0.18 | 0.22 | 0.29 | 0.11 | 0.15 | 0.13 | 0.10 | 0.12 |
| 23 |  | Methyleugenol | 5.89 | 2.59 | 3.36 | 6.80 | 9.85 | 9.43 | 7.00 | 4.78 | 9.15 | 5.55 | 8.92 | 8.25 |
| 24 |  | trans-Isoeugenol | 0.28 | 0.43 | 0.58 | 0.47 | 0.37 | 0.32 | 0.58 | 0.80 | 0.62 | 0.67 | 0.61 | 0.30 |
| 25 |  | Benzene, 1,2-dimethoxy-4-propenyl-, (Z)- | 2.97 | 1.09 | 0.40 | 1.96 | 3.35 | 1.57 | 1.41 | 4.01 | 4.05 | 1.44 | 1.93 | 3.25 |
| 26 |  | 1,3-Benzodioxole, 4-methoxy-6-(2-propenyl)- | 11.08 | 12.69 | 18.15 | 14.19 | 10.67 | 13.06 | 18.27 | 11.36 | 14.88 | 13.51 | 17.91 | 10.47 |
| 27 |  | Benzene,1,2,3-trimethoxy-5-(2-propenyl)- | 2.71 | 5.73 | 4.40 | 4.49 | 4.57 | 5.97 | 4.35 | 3.53 | 5.90 | 4.57 | 7.11 | 4.38 |
| 28 |  | Phenol, 2,6-dimethoxy-4-(2-propenyl)- | 0 | 0 | 0 | 0 | 0 | 0 | 0.11 | 0.16 | 0.14 | 0.13 | 0.10 | 0.31 |
| 29 | Alkanes | Bicyclo[3.1.0]hex-2-ene, 4-methyl-1-(1-methylethyl)- | 2.16 | 1.99 | 1.95 | 1.86 | 1.78 | 2.05 | 1.58 | 1.93 | 1.61 | 1.83 | 1.38 | 1.97 |
| 30 | Alcohols | Bicyclo[3.1.0]hexan-2-ol, 2-methyl-5-(1-methylethyl)-, (1.alpha.,2.beta.,5.alpha.)- | 0.83 | 0.39 | 0.79 | 0.74 | 0.69 | 0.91 | 0.95 | 1.08 | 0.45 | 0.75 | 1.04 | 0.59 |
| 31 |  | Bicyclo[3.1.0]hexan-2-ol, 2-methyl-5-(1-methylethyl)-, (1.alpha.,2.alpha.,5.alpha.)- | 0.74 | 0.32 | 0.7 | 0.65 | 0.60 | 0.81 | 0.84 | 0.90 | 0.38 | 0.63 | 0.97 | 0.52 |
| 32 |  | Linalool | 0.31 | 0.24 | 0.21 | 0.20 | 0.19 | 0.22 | 0.23 | 0.26 | 0.17 | 0.20 | 0.27 | 0.13 |
| 33 |  | 4-Isopropyl-1-methylcyclohex-2-enol | 0.43 | 0.47 | 0.53 | 0.49 | 0.45 | 0.47 | 0.45 | 0.50 | 0.41 | 0.52 | 0.61 | 0.44 |
| 34 |  | 2-Cyclohexen-1-ol, 1-methyl-4-(1-methylethyl)-, cis- | 0.26 | 0.30 | 0.32 | 0.29 | 0.27 | 0.27 | 0.26 | 0.28 | 0.25 | 0.31 | 0.36 | 0.27 |
| 35 |  | 3-Cyclohexen-1-ol, 4-methyl-1-(1-methylethyl)-, (R)- | 7.26 | 8.86 | 9.78 | 9.01 | 8.33 | 8.29 | 8.09 | 8.14 | 7.42 | 10.38 | 11.87 | 8.98 |
| 36 |  | alpha.-Terpineol | 0.63 | 0.73 | 0.71 | 0.69 | 0.67 | 0.64 | 0.65 | 0.67 | 0.64 | 0.74 | 0.91 | 0.57 |
| 37 |  | 2-Cyclohexen-1-ol, 3-methyl-6-(1-methylethyl)-, cis- | 0.12 | 0.14 | 0.12 | 0.12 | 0.13 | 0.10 | 0.10 | 0.11 | 0.09 | 0.11 | 0.13 | 0.13 |
| 38 |  | 2-Cyclohexen-1-ol, 3-methyl-6-(1-methylethyl)-, trans- | 0 | 0 | 0.17 | 0.08 | 0 | 0.13 | 0.11 | 0.14 | 0.11 | 0.14 | 0.14 | 0.38 |
| 39 |  | 5-Azulenemethanol, 1,2,3,4,5,6,7,8-octahydro-.alpha.,.alpha.,3,8-tetramethyl- | 0 | 0 | 0.12 | 0.11 | 0.10 | 0.17 | 0.23 | 0.16 | 0.10 | 0.10 | 0.29 | 0.51 |
| 40 | Aromatic | p-Cymene | 0.67 | 0.90 | 1.05 | 1.04 | 1.04 | 0.91 | 0.79 | 1.19 | 0.91 | 0.80 | 0.70 | 0.86 |
| 41 |  | Benzene, 1-methoxy-4-pentyl- | 0 | 0 | 0 | 0 | 0 | 0 | 0 | 0 | 0 | 0.11 | 0.21 | 0.55 |
| 42 | Esters | Geranyl acetate | 0.34 | 0.14 | 0.1 | 0.10 | 0.10 | 0.14 | 0.13 | 0.14 | 0.10 | 0.12 | 0.16 | 0.11 |
| 43 |  | Tetradecanoi acid, ethyl ester | 0 | 0 | 0 | 0 | 0 | 0.15 | 0.34 | 1.16 | 0.32 | 0.44 | 0.42 | 1.12 |
| 44 | Fatty Acids | Tetradecanoic acid | 0 | 0.13 | 0.17 | 0.08 | 0 | 0.12 | 0 | 0 | 0.23 | 1.92 | 0.54 | 0.90 |

Table 2-1. Peak area of HPLC fingerprint

|  | J0 | J1 | J2 | J3 | J4 | J5 | J6 | J7 | J8 | J9 | J10 | J11 | J12 | J13 | J14 |
| --- | --- | --- | --- | --- | --- | --- | --- | --- | --- | --- | --- | --- | --- | --- | --- |
| cpd1 | 1.902 | 1.333 | 0 | 8.318 | 1.107 | 0 | 0 | 0 | 3.189 | 4.628 | 0 | 1.848 | 0 | 0 | 0 |
| cpd2 | 0 | 9.523 | 0 | 60.942 | 0 | 0 | 18.205 | 15.599 | 2.779 | 0 | 0 | 0 | 0 | 7.9 | 0 |
| cpd3 | 39.386 | 47.937 | 80.941 | 0 | 80.925 | 140.526 | 127.764 | 81.076 | 70.71 | 88.095 | 121.023 | 147.103 | 143.771 | 82.782 | 54.96 |
| cpd4 | 61.942 | 98.551 | 85.686 | 69.413 | 96.078 | 99.756 | 107.727 | 119.401 | 92.246 | 74.547 | 96.705 | 131.039 | 105.2 | 74.623 | 42.663 |
| cpd5 | 0 | 0 | 0 | 0 | 0 | 358.027 | 0 | 511.642 | 0 | 409.619 | 0 | 727.65 | 377.914 | 0 | 28.028 |
| cpd6 | 9.041 | 53.475 | 18.21 | 0 | 60.757 | 0 | 87.449 | 0 | 43.185 | 0 | 370.562 | 0 | 0 | 261.286 | 81.811 |
| cpd7 | 0 | 9.569 | 0 | 0 | 8.495 | 10.583 | 2.524 | 0 | 5.459 | 0 | 0 | 0 | 0 | 0 | 0 |
| cpd8 | 8.883 | 33.878 | 19.965 | 0 | 30.961 | 0 | 7.556 | 0 | 25.118 | 8.173 | 11.974 | 3.178 | 19.546 | 4.523 | 21.58 |
| cpd9 | 0 | 4.438 | 0 | 0 | 0 | 0 | 0 | 0 | 0 | 0 | 0 | 0 | 0 | 0 | 0 |
| cpd10 | 5.442 | 10.245 | 8.257 | 19.636 | 6.246 | 7.71 | 8.89 | 14.642 | 4.954 | 13.812 | 5.905 | 8.9 | 7.458 | 14.294 | 6.167 |
| cpd11 | 0 | 3.114 | 0 | 0 | 2.597 | 0 | 0 | 0 | 4.153 | 0 | 3.819 | 4.32 | 0 | 0 | 0 |
| cpd12 | 9.066 | 5.432 | 0 | 0 | 3.638 | 6.03 | 3.822 | 9.615 | 0 | 4.678 | 6.863 | 3.173 | 11.3 | 10.725 | 3.677 |
| cpd13 | 0 | 10.224 | 0 | 0 | 0 | 0 | 0 | 0 | 0 | 0 | 0 | 0 | 9.581 | 0 | 0 |
| cpd14 | 0 | 0 | 0 | 0 | 0 | 0 | 0 | 0 | 0 | 4.345 | 0 | 4.652 | 0 | 0 | 0 |
| cpd15 | 0 | 0 | 0 | 0 | 0 | 0 | 0 | 0 | 0 | 0 | 0 | 0 | 0 | 0 | 0 |
| cpd16 | 0 | 0 | 0 | 13.755 | 10.826 | 16.337 | 10.04 | 19.879 | 0 | 0 | 47.535 | 0 | 12.066 | 46.17 | 0 |
| cpd17 | 7.12 | 16.638 | 11.985 | 0 | 0 | 0 | 0 | 0 | 44.965 | 20.146 | 0 | 12.817 | 0 | 26.649 | 0 |
| cpd18 | 0 | 17.139 | 5.67 | 11.011 | 3.545 | 10.065 | 16.938 | 10.756 | 9.19 | 15.275 | 15.572 | 12.701 | 9.893 | 61.553 | 8.448 |
| cpd19 | 0 | 0 | 0 | 0 | 0 | 0 | 5.458 | 7.415 | 5.499 | 4.287 | 4.228 | 0 | 0 | 11.963 | 0 |
| cpd20 | 0 | 0 | 0 | 0 | 0 | 0 | 0 | 0 | 0 | 0 | 0 | 0 | 0 | 0 | 0 |
| cpd21 | 6.088 | 30.78 | 36.21 | 0 | 6.104 | 0 | 12.947 | 10.862 | 20.053 | 10.07 | 11.49 | 18.014 | 26.639 | 31.324 | 18.16 |
| cpd22 | 0 | 0 | 0 | 0 | 0 | 0 | 0 | 0 | 0 | 0 | 0 | 0 | 0 | 0 | 112.365 |
| cpd23 | 80.166 | 108.015 | 82.952 | 8.512 | 77.339 | 82 | 69.425 | 50.054 | 83.695 | 119.745 | 103.679 | 51.707 | 152.1 | 108.665 | 0 |
| cpd24 | 0 | 0 | 0 | 0 | 0 | 0 | 0 | 0 | 0 | 0 | 0 | 0 | 0 | 0 | 101.995 |
| cpd25 | 0 | 0 | 0 | 15.291 | 0 | 11.935 | 102.527 | 77.682 | 0 | 0 | 30.235 | 0 | 123.23 | 204.272 | 0 |
| cpd26 | 13.208 | 17.011 | 24.376 | 0 | 0 | 0 | 0 | 0 | 17.61 | 97.147 | 0 | 8.676 | 0 | 0 | 0 |
| cpd27 | 203.629 | 716.529 | 322.992 | 16.445 | 841.742 | 61.584 | 759.526 | 32.777 | 613.696 | 45.992 | 407.943 | 36.382 | 609.417 | 77.17 | 195.86 |
| cpd28 | 133.426 | 0 | 0 | 0 | 0 | 0 | 0 | 15.709 | 0 | 14.253 | 0 | 0 | 0 | 27.455 | 0 |
| cpd29 | 389.088 | 219.141 | 434.547 | 6.649 | 844.186 | 29.344 | 262.016 | 9.447 | 596.404 | 5.312 | 385.428 | 7.914 | 304.204 | 26.785 | 373.249 |
| cpd30 | 0 | 0 | 0 | 0 | 0 | 0 | 0 | 0 | 0 | 0 | 0 | 0 | 7.249 | 0 | 0 |
| cpd31 | 0 | 0 | 0 | 0 | 24.516 | 0 | 0 | 0 | 0 | 0 | 0 | 0 | 0 | 0 | 0 |
| cpd32 | 0 | 28.279 | 0 | 0 | 0 | 0 | 0 | 0 | 0 | 0 | 0 | 0 | 22.066 | 10.748 | 0 |
| cpd33 | 15.629 | 0 | 0 | 0 | 0 | 0 | 0 | 0 | 0 | 0 | 6.433 | 0 | 0 | 0 | 0 |
| cpd34 | 0 | 0 | 0 | 0 | 0 | 0 | 0 | 10.031 | 0 | 10.057 | 0 | 0 | 0 | 35.357 | 0 |
| cpd35 | 27.207 | 0 | 36.12 | 0 | 38.973 | 0 | 0 | 27.594 | 22.988 | 24.22 | 38.447 | 8.149 | 34.538 | 0 | 0 |
| cpd36 | 74.848 | 90.035 | 109.947 | 99.904 | 102.239 | 115.399 | 69.414 | 160.703 | 55.885 | 115.903 | 122.446 | 147.721 | 124.593 | 247.913 | 57.074 |
| cpd37 | 0 | 0 | 0 | 0 | 0 | 0 | 0 | 0 | 0 | 0 | 0 | 0 | 0 | 0 | 353.231 |
| cpd38 | 105.394 | 200.331 | 288.265 | 445.655 | 142.092 | 389.098 | 490.251 | 396.764 | 194.755 | 442.138 | 572.102 | 381.053 | 417.038 | 1087.865 | 0 |
| cpd39 | 0 | 0 | 0 | 0 | 15.819 | 0 | 0 | 0 | 0 | 0 | 0 | 0 | 0 | 28.766 | 32.188 |
| cpd40 | 0 | 0 | 0 | 0 | 0 | 0 | 18.777 | 36.377 | 0 | 18.548 | 18.646 | 0 | 29.742 | 0 | 0 |
| cpd41 | 0 | 0 | 0 | 0 | 0 | 0 | 0 | 0 | 0 | 0 | 0 | 0 | 0 | 0 | 19.682 |
| cpd42 | 30.245 | 36.306 | 21.113 | 35.175 | 37.584 | 40.301 | 26.827 | 72.851 | 19.403 | 42.306 | 57.942 | 30.594 | 35.432 | 107.059 | 0 |
| cpd43 | 0 | 0 | 13.08 | 0 | 0 | 0 | 12.296 | 24.165 | 0 | 0 | 0 | 0 | 13.021 | 0 | 11.699 |
| cpd44 | 16.726 | 62.929 | 22.156 | 52.754 | 27.437 | 92.244 | 125.419 | 84.835 | 48.324 | 74.111 | 121.22 | 93.747 | 101.729 | 188.071 | 84.485 |
| cpd45 | 0 | 17.501 | 0 | 0 | 0 | 0 | 0 | 0 | 0 | 0 | 19.935 | 16.172 | 0 | 6.84 | 0 |
| cpd46 | 46.287 | 0 | 0 | 0 | 0 | 0 | 0 | 0 | 0 | 0 | 0 | 0 | 0 | 0 | 74.294 |
| cpd47 | 0 | 0 | 0 | 0 | 54.959 | 0 | 0 | 0 | 0 | 0 | 0 | 0 | 0 | 0 | 0 |
| cpd48 | 172.222 | 172.087 | 120.327 | 41.544 | 0 | 61.923 | 99.904 | 92.858 | 88.967 | 101.055 | 152.689 | 109.876 | 143.099 | 228.376 | 0 |
| cpd49 | 0 | 0 | 0 | 0 | 0 | 0 | 0 | 13.619 | 0 | 0 | 48.652 | 0 | 8.987 | 22.084 | 23.172 |
| cpd50 | 0 | 0 | 0 | 0 | 0 | 0 | 0 | 0 | 0 | 0 | 0 | 0 | 0 | 0 | 0 |
| cpd51 | 0 | 0 | 0 | 0 | 8.364 | 8.446 | 0 | 0 | 0 | 0 | 15.729 | 0 | 13.288 | 0 | 66.325 |
| cpd52 | 0 | 0 | 0 | 0 | 0 | 0 | 0 | 0 | 0 | 4.791 | 0 | 0 | 0 | 208.308 | 0 |
| cpd53 | 46.841 | 98.297 | 65.408 | 53.398 | 46.089 | 92.312 | 90.872 | 84.961 | 53.426 | 84.132 | 107.975 | 72.655 | 94.129 | 0 | 0 |
| cpd54 | 48.51 | 0 | 0 | 0 | 0 | 14.944 | 12.479 | 0 | 13.254 | 0 | 24.452 | 0 | 0 | 14.625 | 39.784 |
| cpd55 | 20.109 | 0 | 54.979 | 29.575 | 0 | 23.379 | 56.453 | 114.135 | 0 | 0 | 0 | 0 | 13.278 | 0 | 192.684 |
| cpd56 | 0 | 0 | 0 | 0 | 0 | 0 | 0 | 0 | 0 | 0 | 0 | 0 | 0 | 0 | 0 |
| cpd57 | 0 | 0 | 0 | 0 | 54.744 | 0 | 0 | 0 | 0 | 0 | 0 | 0 | 0 | 98.561 | 20.544 |
| cpd58 | 0 | 47.479 | 61.01 | 0 | 0 | 60.249 | 35.466 | 44.947 | 100.118 | 59.808 | 125.888 | 58.361 | 51.385 | 0 | 100.274 |
| cpd59 | 80.255 | 0 | 0 | 0 | 47.337 | 0 | 0 | 0 | 0 | 0 | 0 | 0 | 0 | 213.002 | 0 |
| cpd60 | 227.836 | 0 | 220.809 | 81.706 | 0 | 59.085 | 328.243 | 0 | 191.313 | 66.512 | 79.222 | 129.299 | 164.857 | 0 | 0 |
| cpd61 | 0 | 0 | 0 | 0 | 0 | 48.098 | 0 | 0 | 0 | 0 | 0 | 0 | 0 | 0 | 0 |
| cpd62 | 0 | 0 | 0 | 0 | 0 | 0 | 0 | 0 | 0 | 0 | 0 | 0 | 0 | 0 | 164.84 |
| cpd63 | 388.798 | 371.42 | 440.225 | 145.769 | 284.635 | 308.893 | 319.943 | 312.195 | 536.507 | 231.406 | 643.474 | 373.542 | 468.282 | 776.736 | 0 |
| cpd64 | 0 | 0 | 0 | 0 | 0 | 0 | 14.415 | 0 | 0 | 0 | 0 | 0 | 0 | 38.069 | 13.643 |
| cpd65 | 38.13 | 0 | 0 | 0 | 0 | 0 | 0 | 0 | 0 | 0 | 0 | 0 | 0 | 0 | 0 |
| cpd66 | 13.843 | 12.067 | 0 | 0 | 0 | 0 | 0 | 17.09 | 0 | 10.587 | 15.035 | 11.408 | 0 | 25.139 | 16.144 |
| cpd67 | 16.061 | 0 | 0 | 0 | 0 | 0 | 0 | 0 | 0 | 0 | 0 | 0 | 0 | 0 | 137.746 |
| cpd68 | 270.022 | 347.104 | 345.614 | 109.069 | 253.899 | 171.552 | 406.031 | 149.068 | 344.01 | 168.06 | 292.207 | 201.719 | 338.194 | 298.585 | 0 |
| cpd69 | 0 | 0 | 0 | 0 | 0 | 0 | 0 | 0 | 0 | 0 | 0 | 0 | 0 | 36.769 | 14.405 |
| cpd70 | 0 | 0 | 0 | 0 | 0 | 9.568 | 27.707 | 0 | 10.288 | 0 | 0 | 0 | 33.271 | 0 | 0 |
| cpd71 | 65.575 | 0 | 0 | 0 | 0 | 0 | 0 | 0 | 0 | 0 | 0 | 0 | 0 | 43.53 | 27.124 |
| cpd72 | 108.897 | 45.586 | 30.853 | 0 | 13.199 | 0 | 15.063 | 0 | 45.048 | 10.229 | 17.523 | 15.742 | 34.141 | 0 | 0 |
| cpd73 | 0 | 0 | 0 | 0 | 0 | 0 | 0 | 0 | 0 | 0 | 131.95 | 0 | 0 | 0 | 0 |
| cpd74 | 5.136 | 0 | 0 | 0 | 0 | 0 | 0 | 0 | 0 | 0 | 0 | 0 | 0 | 0 | 0 |
| cpd75 | 0 | 0 | 0 | 0 | 0 | 0 | 0 | 0 | 0 | 0 | 0 | 0 | 0 | 0 | 66.519 |
| cpd76 | 147.472 | 147.682 | 132.888 | 142.12 | 140.009 | 133.918 | 121.446 | 129.356 | 124.237 | 148.177 | 0 | 144.717 | 116.059 | 73.063 | 0 |
| cpd77 | 0 | 0 | 0 | 0 | 0 | 0 | 0 | 0 | 0 | 0 | 0 | 0 | 0 | 0 | 19.325 |
| cpd78 | 0 | 0 | 0 | 63.507 | 47.422 | 0 | 0 | 0 | 0 | 0 | 67.883 | 0 | 51.51 | 37.603 | 0 |
| cpd79 | 47.849 | 33.056 | 52.515 | 0 | 0 | 70.012 | 53.251 | 24.233 | 49.525 | 24.776 | 0 | 27.51 | 0 | 0 | 0 |
| cpd80 | 1432.338 | 193.142 | 268.052 | 107.009 | 276.51 | 153.962 | 33.911 | 225.629 | 448.708 | 239.734 | 235.434 | 99.469 | 161.072 | 377.542 | 86.823 |
| cpd81 | 703.983 | 432.066 | 327.546 | 143.876 | 205.802 | 268.205 | 83.584 | 408.923 | 475.179 | 296.637 | 476.167 | 253.629 | 112.167 | 313.036 | 96.964 |
| cpd82 | 111.052 | 83.094 | 245.226 | 62.26 | 200.228 | 114.679 | 227.011 | 114.415 | 86.743 | 111.322 | 261.36 | 184.13 | 147.914 | 174.552 | 69.278 |
| cpd83 | 0 | 0 | 0 | 0 | 0 | 0 | 0 | 0 | 0 | 0 | 0 | 0 | 0 | 25.309 | 0 |
| cpd84 | 0 | 0 | 0 | 0 | 29.752 | 0 | 0 | 0 | 0 | 0 | 42.28 | 0 | 14.379 | 0 | 0 |
| cpd85 | 0 | 9.321 | 52.403 | 0 | 0 | 17.537 | 26.498 | 10.157 | 0 | 0 | 0 | 15.961 | 0 | 0 | 0 |
| cpd86 | 35.278 | 0 | 0 | 37.918 | 33.089 | 0 | 0 | 0 | 0 | 0 | 0 | 0 | 0 | 0 | 0 |
| cpd87 | 22.245 | 28.577 | 31.295 | 0 | 0 | 35.925 | 39.741 | 40.259 | 0 | 32.115 | 0 | 16.228 | 23.121 | 0 | 0 |
| cpd88 | 0 | 0 | 10.281 | 0 | 0 | 10.216 | 0 | 0 | 0 | 0 | 0 | 0 | 65.402 | 0 | 0 |
| cpd89 | 0 | 0 | 0 | 0 | 17.483 | 29.538 | 0 | 0 | 0 | 0 | 0 | 0 | 14.659 | 0 | 0 |
| cpd90 | 58.771 | 0 | 102.455 | 203.694 | 0 | 0 | 28.213 | 20.975 | 0 | 0 | 0 | 0 | 37.67 | 0 | 45.055 |
| cpd91 | 0 | 0 | 0 | 0 | 0 | 0 | 0 | 0 | 0 | 0 | 0 | 0 | 40.329 | 0 | 0 |
| cpd92 | 0 | 0 | 0 | 0 | 0 | 14.018 | 0 | 0 | 0 | 0 | 0 | 0 | 43.398 | 0 | 0 |
| cpd93 | 0 | 0 | 0 | 0 | 0 | 0 | 0 | 0 | 0 | 0 | 0 | 0 | 0 | 0 | 0 |

Table 2-2. Peak area of HPLC fingerprint

|  | J15 | J16 | J17 | J18 | J19 | J20 | J21 | J22 | J23 | J24 | J25 | J26 |
| --- | --- | --- | --- | --- | --- | --- | --- | --- | --- | --- | --- | --- |
| cpd1 | 0 | 0 | 0 | 0 | 0 | 0 | 7.905 | 5.301 | 10.181 | 6.805 | 10.742 | 3.87 |
| cpd2 | 0 | 0 | 0 | 7.685 | 0 | 0 | 132.477 | 0 | 0 | 0 | 0 | 0 |
| cpd3 | 55.104 | 111.671 | 140.278 | 66.792 | 69.019 | 105.126 | 0 | 176.299 | 133.353 | 195.06 | 69.937 | 359.132 |
| cpd4 | 60.944 | 68.124 | 95.467 | 96.958 | 43.435 | 44.577 | 66.858 | 129.771 | 115.183 | 162.414 | 85.438 | 181.64 |
| cpd5 | 0 | 15.564 | 0 | 0 | 0 | 0 | 0 | 0 | 0 | 0 | 0 | 0 |
| cpd6 | 69.017 | 23.538 | 114.2 | 45.869 | 24.415 | 76.399 | 266.553 | 74.657 | 82.384 | 183.95 | 0 | 0 |
| cpd7 | 16.544 | 10.775 | 15.535 | 7.32 | 0 | 11.524 | 0 | 0 | 7.711 | 0 | 0 | 54.367 |
| cpd8 | 50.099 | 11.516 | 13.03 | 14.273 | 3.045 | 0 | 0 | 0 | 0 | 0 | 0 | 20.863 |
| cpd9 | 4.242 | 0 | 0 | 0 | 0 | 0 | 0 | 0 | 0 | 0 | 0 | 0 |
| cpd10 | 0 | 14.24 | 4.078 | 11.196 | 16.725 | 7.424 | 0 | 4.739 | 0 | 0 | 16.532 | 0 |
| cpd11 | 2.571 | 0 | 3.816 | 5.366 | 4.987 | 0 | 0 | 0 | 0 | 0 | 0 | 12.836 |
| cpd12 | 0 | 6.714 | 0 | 6.317 | 16.899 | 0 | 11.474 | 4.656 | 10.673 | 16.614 | 3.655 | 4.602 |
| cpd13 | 0 | 0 | 0 | 0 | 0 | 0 | 0 | 0 | 0 | 0 | 12.076 | 0 |
| cpd14 | 0 | 0 | 0 | 0 | 0 | 15.772 | 0 | 0 | 0 | 0 | 0 | 0 |
| cpd15 | 0 | 0 | 0 | 0 | 0 | 0 | 45.507 | 4.985 | 9.9 | 48.995 | 10.22 | 76.881 |
| cpd16 | 21.433 | 15.054 | 12.294 | 14.205 | 21.604 | 15.741 | 0 | 0 | 0 | 0 | 0 | 0 |
| cpd17 | 0 | 0 | 15.138 | 0 | 0 | 0 | 0 | 0 | 0 | 0 | 14.605 | 0 |
| cpd18 | 9.567 | 6.673 | 17.76 | 12.198 | 7.941 | 0 | 0 | 0 | 0 | 0 | 8.781 | 6.329 |
| cpd19 | 4.794 | 0 | 5.841 | 14.434 | 0 | 0 | 0 | 0 | 0 | 0 | 0 | 9.42 |
| cpd20 | 0 | 0 | 0 | 0 | 0 | 9.194 | 0 | 0 | 0 | 17.35 | 0 | 13.744 |
| cpd21 | 13.203 | 0 | 12.082 | 0 | 29.758 | 0 | 0 | 0 | 0 | 0 | 0 | 0 |
| cpd22 | 0 | 20.916 | 0 | 0 | 93.721 | 37.602 | 57.325 | 75.518 | 131.821 | 63.664 | 62.948 | 57.295 |
| cpd23 | 54.087 | 49.08 | 57.865 | 60.047 | 0 | 0 | 0 | 0 | 0 | 0 | 0 | 0 |
| cpd24 | 0 | 0 | 12.51 | 0 | 76.823 | 0 | 12.989 | 0 | 62.99 | 0 | 105.281 | 25.513 |
| cpd25 | 10.094 | 31.29 | 0 | 6.824 | 0 | 32.498 | 0 | 0 | 0 | 0 | 0 | 0 |
| cpd26 | 0 | 0 | 0 | 0 | 0 | 0 | 0 | 0 | 0 | 0 | 0 | 0 |
| cpd27 | 1428.489 | 304.486 | 1158.4 | 1141.365 | 57.298 | 415.879 | 13.18 | 33.737 | 96.637 | 5.179 | 29.215 | 1065.181 |
| cpd28 | 0 | 0 | 0 | 0 | 15.284 | 0 | 0 | 0 | 0 | 0 | 0 | 0 |
| cpd29 | 1069.624 | 374.431 | 389.898 | 390.354 | 15.671 | 706.628 | 7.958 | 7.424 | 6.304 | 9.976 | 0 | 1647.523 |
| cpd30 | 0 | 0 | 0 | 0 | 16.592 | 0 | 0 | 0 | 0 | 0 | 13.491 | 0 |
| cpd31 | 29.314 | 0 | 0 | 0 | 0 | 0 | 5.227 | 0 | 0 | 0 | 0 | 0 |
| cpd32 | 0 | 18.491 | 0 | 0 | 0 | 17.946 | 17.714 | 0 | 0 | 0 | 0 | 36.73 |
| cpd33 | 0 | 0 | 16.874 | 4.793 | 0 | 0 | 0 | 0 | 20.856 | 170.075 | 0 | 0 |
| cpd34 | 0 | 0 | 0 | 0 | 0 | 0 | 0 | 0 | 0 | 0 | 0 | 0 |
| cpd35 | 0 | 44.806 | 0 | 22.027 | 0 | 34.764 | 0 | 0 | 0 | 0 | 0 | 0 |
| cpd36 | 78.311 | 173.196 | 95.095 | 128.709 | 228.747 | 103.467 | 171.054 | 38.534 | 249.233 | 196.115 | 104.911 | 213.112 |
| cpd37 | 0 | 0 | 215.061 | 0 | 462.5 | 0 | 196.443 | 635.479 | 133.931 | 465.49 | 797.725 | 515.962 |
| cpd38 | 105.687 | 538.817 | 0 | 182.918 | 0 | 107.596 | 0 | 0 | 0 | 0 | 0 | 0 |
| cpd39 | 22.149 | 0 | 15.908 | 0 | 30.382 | 0 | 0 | 0 | 0 | 0 | 0 | 0 |
| cpd40 | 0 | 35.764 | 0 | 18.219 | 0 | 20.164 | 0 | 0 | 0 | 0 | 0 | 0 |
| cpd41 | 0 | 0 | 45.583 | 0 | 34.955 | 0 | 17.452 | 8.978 | 20.495 | 33.273 | 30.558 | 36.595 |
| cpd42 | 41.367 | 53.704 | 0 | 32.856 | 0 | 21.186 | 0 | 0 | 0 | 0 | 0 | 0 |
| cpd43 | 19.757 | 33.203 | 0 | 0 | 0 | 0 | 0 | 0 | 24.741 | 0 | 0 | 0 |
| cpd44 | 19.861 | 74.846 | 24.577 | 27.951 | 84.865 | 11.083 | 33.682 | 89.545 | 16.111 | 42.439 | 104.672 | 46.654 |
| cpd45 | 0 | 0 | 0 | 0 | 10.011 | 0 | 0 | 0 | 0 | 8.176 | 0 | 0 |
| cpd46 | 47.094 | 0 | 0 | 0 | 0 | 0 | 0 | 0 | 202.522 | 242.463 | 106.082 | 0 |
| cpd47 | 0 | 71.176 | 98.315 | 60.661 | 248.377 | 90.41 | 150.354 | 43.856 | 0 | 0 | 0 | 55.345 |
| cpd48 | 0 | 0 | 0 | 0 | 0 | 0 | 0 | 0 | 0 | 0 | 0 | 0 |
| cpd49 | 0 | 0 | 0 | 0 | 0 | 0 | 0 | 0 | 0 | 38.56 | 0 | 0 |
| cpd50 | 0 | 0 | 0 | 0 | 13.306 | 0 | 0 | 0 | 18.637 | 0 | 0 | 6.779 |
| cpd51 | 0 | 20.927 | 53.595 | 11.903 | 129.24 | 17.606 | 62.405 | 29.955 | 53.033 | 59.143 | 52.85 | 65.748 |
| cpd52 | 55.251 | 0 | 0 | 0 | 0 | 60.121 | 0 | 0 | 0 | 0 | 0 | 0 |
| cpd53 | 0 | 55.824 | 0 | 112.745 | 0 | 0 | 0 | 0 | 0 | 0 | 0 | 0 |
| cpd54 | 18.371 | 22.648 | 0 | 0 | 18.675 | 26.874 | 0 | 0 | 17.961 | 36.983 | 0 | 0 |
| cpd55 | 133.388 | 170.894 | 44.205 | 0 | 0 | 76.756 | 0 | 0 | 130.257 | 0 | 0 | 0 |
| cpd56 | 0 | 0 | 44.907 | 0 | 77.851 | 0 | 0 | 0 | 14.35 | 0 | 0 | 53.801 |
| cpd57 | 0 | 0 | 0 | 0 | 0 | 0 | 0 | 0 | 0 | 0 | 0 | 0 |
| cpd58 | 0 | 0 | 0 | 39.819 | 290.674 | 0 | 495.723 | 0 | 21.765 | 662.617 | 377.182 | 0 |
| cpd59 | 539.141 | 0 | 0 | 0 | 0 | 555.352 | 0 | 0 | 0 | 0 | 0 | 0 |
| cpd60 | 0 | 523.057 | 0 | 0 | 0 | 0 | 0 | 0 | 0 | 0 | 0 | 0 |
| cpd61 | 0 | 0 | 0 | 0 | 0 | 0 | 0 | 0 | 0 | 0 | 0 | 0 |
| cpd62 | 0 | 0 | 279.741 | 0 | 692.621 | 0 | 1072.778 | 33.365 | 277.218 | 1083.656 | 138.409 | 889.148 |
| cpd63 | 472.634 | 665.396 | 0 | 292.598 | 0 | 426.851 | 0 | 0 | 0 | 0 | 0 | 0 |
| cpd64 | 0 | 0 | 25.248 | 0 | 24.573 | 30.083 | 37.819 | 0 | 0 | 0 | 0 | 0 |
| cpd65 | 0 | 0 | 0 | 0 | 0 | 0 | 0 | 0 | 0 | 0 | 0 | 0 |
| cpd66 | 0 | 0 | 0 | 9.381 | 0 | 0 | 0 | 0 | 0 | 0 | 0 | 0 |
| cpd67 | 0 | 0 | 327.012 | 0 | 395.192 | 0 | 235.696 | 118.16 | 345.189 | 409.477 | 211.298 | 389.62 |
| cpd68 | 158.272 | 143.012 | 0 | 266.008 | 0 | 248.698 | 0 | 0 | 0 | 0 | 0 | 0 |
| cpd69 | 0 | 0 | 0 | 0 | 12.751 | 0 | 19.386 | 0 | 0 | 19.99 | 0 | 0 |
| cpd70 | 0 | 0 | 0 | 0 | 0 | 0 | 0 | 0 | 0 | 0 | 0 | 0 |
| cpd71 | 16.63 | 0 | 0 | 0 | 23.318 | 0 | 0 | 0 | 0 | 0 | 0 | 69.82 |
| cpd72 | 0 | 0 | 0 | 10.03 | 0 | 0 | 0 | 0 | 0 | 0 | 0 | 0 |
| cpd73 | 0 | 0 | 0 | 0 | 0 | 0 | 0 | 0 | 18.367 | 0 | 0 | 0 |
| cpd74 | 0 | 8.24 | 0 | 0 | 0 | 0 | 0 | 0 | 0 | 0 | 0 | 0 |
| cpd75 | 0 | 0 | 161.257 | 0 | 62.04 | 0 | 195.54 | 162.978 | 41.785 | 36.16 | 36.367 | 35.472 |
| cpd76 | 130.883 | 81.607 | 0 | 148.279 | 0 | 53.402 | 0 | 0 | 106.093 | 0 | 0 | 0 |
| cpd77 | 0 | 0 | 62.678 | 0 | 63.872 | 0 | 62.951 | 32.752 | 282.977 | 80.143 | 54.351 | 64.834 |
| cpd78 | 28.523 | 29.543 | 0 | 0 | 0 | 46.663 | 0 | 0 | 0 | 0 | 0 | 0 |
| cpd79 | 0 | 0 | 0 | 62.182 | 0 | 0 | 0 | 0 | 0 | 0 | 0 | 0 |
| cpd80 | 39.302 | 57.657 | 242.67 | 229.948 | 82.084 | 41.327 | 181.459 | 24.915 | 569.554 | 1119.243 | 46.044 | 511.125 |
| cpd81 | 84.79 | 50.954 | 440.332 | 363.122 | 118.344 | 92.714 | 196.679 | 26.038 | 393.264 | 806.817 | 45.361 | 928.767 |
| cpd82 | 226.362 | 202.768 | 206.179 | 159.993 | 147.412 | 207.099 | 132.365 | 192.026 | 503.52 | 221.99 | 181.07 | 147.029 |
| cpd83 | 0 | 0 | 9.192 | 0 | 17.246 | 28.826 | 0 | 0 | 57.51 | 0 | 0 | 0 |
| cpd84 | 14.505 | 14.578 | 0 | 0 | 0 | 0 | 0 | 14.634 | 0 | 0 | 38.173 | 0 |
| cpd85 | 0 | 0 | 21.471 | 19.992 | 0 | 0 | 12.749 | 0 | 0 | 0 | 0 | 13.411 |
| cpd86 | 33.313 | 0 | 0 | 0 | 0 | 0 | 0 | 0 | 0 | 0 | 0 | 0 |
| cpd87 | 0 | 0 | 0 | 29.246 | 0 | 0 | 0 | 0 | 0 | 0 | 0 | 0 |
| cpd88 | 0 | 11.971 | 0 | 0 | 20.281 | 0 | 28.701 | 0 | 0 | 46.467 | 57.838 | 0 |
| cpd89 | 0 | 11.051 | 0 | 0 | 16.617 | 9.715 | 19.574 | 0 | 0 | 175.898 | 198.575 | 0 |
| cpd90 | 33.423 | 47.802 | 11.386 | 0 | 0 | 231.163 | 16.536 | 0 | 321.505 | 0 | 0 | 0 |
| cpd91 | 0 | 0 | 0 | 0 | 0 | 0 | 0 | 0 | 0 | 0 | 0 | 0 |
| cpd92 | 0 | 18.804 | 0 | 0 | 10.951 | 28.493 | 28.752 | 0 | 0 | 72.907 | 53.971 | 0 |
| cpd93 | 0 | 0 | 0 | 0 | 0 | 0 | 0 | 0 | 3.789 | 0 | 0 | 0 |
